# Cumulative Burden of Fatty Liver and Kidney Cancer in Young Men: A National Population-Based Study

**DOI:** 10.3390/jcm14010148

**Published:** 2024-12-30

**Authors:** Hee Yeon Lee, Kyung Do Han, Hyuk-Sang Kwon

**Affiliations:** 1 Division of Oncology, Department of Internal Medicine, Yeouido St. Mary’s Hospital, College of Medicine, The Catholic University of Korea, Seoul 07345, Republic of Korea; urloved@catholic.ac.kr; 2 Department of Statistics and Actuarial Science, Soongsil University, 369, Sangdo-ro, Dongjak-gu, Seoul 06978, Republic of Korea; 3 Division of Endocrinology and Metabolism, Department of Internal Medicine, Yeouido St. Mary’s Hospital, College of Medicine, The Catholic University of Korea, 10, 63-ro, Yeongdeungpo-gu, Seoul 07345, Republic of Korea

**Keywords:** non-alcoholic fatty liver disease, kidney neoplasms, young adult

## Abstract

**Background:** This national population-based study aimed to assess the cumulative burden of non-alcoholic fatty liver disease (NAFLD) measured via the fatty liver index (FLI) and its association with kidney cancer risk in young men aged 20–39. **Methods**: Using the Korean National Health Insurance Service database, we examined a cohort of 1,007,906 men (age 20–39) who underwent four consecutive annual check-ups from 2009 to 2012. The FLI, calculated from body mass index values, waist circumference, triglyceride levels, and gamma-glutamyl transferase levels, was used to quantify the cumulative burden of NAFLD (FLI ≥ 60). The study population was followed until a kidney cancer diagnosis was made, death occurred, or the advent of 2020. **Results:** Over a mean follow-up of 7.74 years, 649 subjects developed kidney cancer. An increasing mean FLI was associated with an elevated hazard ratio (HR) for kidney cancer. The cumulative frequency of NAFLD (0–4) showed a corresponding increase in the HR for kidney cancer. This association persisted after adjusting for lifestyle factors including smoking, alcohol consumption, and physical activity. Subjects with improved NAFLD had a decreased risk of kidney cancer compared to those with persistent or aggravated NAFLD. **Conclusions:** This study revealed a significant association between NAFLD and kidney cancer in young men. Addressing NAFLD may offer a valuable opportunity to mitigate premature morbidity and mortality associated with young-onset kidney cancer in subsequent generations.

## 1. Introduction

Kidney cancer is the 14th most common cancer worldwide, with 430,000 new cases diagnosed in 2020. Kidney cancer is associated with metabolic diseases due to its links with metabolic risk factors such as obesity and metabolic syndrome. We previously reported an association between kidney cancer and components of metabolic syndrome, noting a stronger link in young males [1]. Recent trends indicate a rising incidence of kidney cancer, along with increasing health care costs due to the development of new treatments. Given these factors, identifying modifiable risk factors is a valuable objective from a public health perspective.

Associations between fatty liver disease and cancer have been reported in several studies [2,3,4]. The pathophysiology involves an inflammatory state and insulin resistance. The fatty liver index (FLI) is a useful and noninvasive algorithm for predicting non-alcoholic fatty liver disease (NAFLD). It is calculated based on four variables: body mass index (BMI), waist circumference (WC), triglyceride (TG) levels, and gamma-glutamyl transferase (GGT) levels [5]. The FLI has been shown to have a high accuracy rate in detecting NAFLD. It has been validated through its application to large populations [6,7]. The FLI ranges from 0 to 100. An FLI < 30 excludes the presence of hepatic steatosis, and an FLI ≥ 60 indicates the presence of disease as detected via ultrasonography [5].

This study aimed to assess the association between the cumulative burden of fatty liver, as measured using the FLI, and the risk of kidney cancer among young men.

## 2. Methods

### 2.1. Data Source

A population-based cohort was established using data from the Korean National Health Insurance Service (NHIS) database. The NHIS is a government-managed national health insurance system that covers nearly 97% of the entire population [8]. The NHIS provides a health-screening program for all insured adults or employees, and approximately 76% of the target population participates in this program. The NHIS database provides comprehensive patient-related data, including demographics, anthropometric measurements, lifestyle survey responses, laboratory results, and diagnostic codes based on the International Classification of Diseases, 10th Revision, Clinical Modification (ICD-10-CM).

### 2.2. Study Population

**Figure 1:**
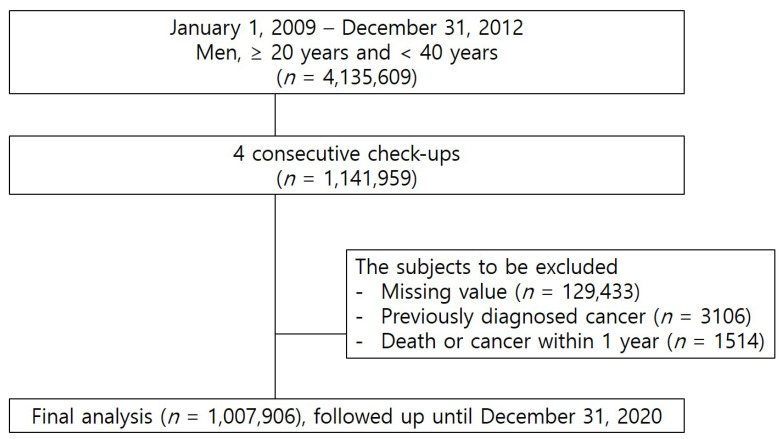
Study design.

A total of 1,007,906 men aged 20 to 39 who underwent four consecutive annual check-ups from January 2009 to December 2012 were analyzed. Individuals with missing data, a history of malignancy, or who were diagnosed with cancer or died within one year after their last check-up were excluded. Participants were followed until a diagnosis of kidney cancer was made, death had occurred, or December 2020, whichever occurred first. Figure 1 shows the study design. This study was conducted according to the guidelines established in the Declaration of Helsinki. It was approved by the Institutional Review Board (IRB) of the Catholic University of Korea (IRB No.SC23ZASI0003). Due to our exclusive use of anonymized and deidentified data in this study, the requirement for informed consent was waived by the IRB. This study followed the Reporting of studies Conducted using Observational Routinely collected health Data (RECORD) guidelines.

### 2.3. Definition

Subjects with kidney cancer were defined as having an ICD-10 code of C64. Subjects with other ‘C’ codes were excluded. Self-reported lifestyle variables including smoking status (non-smoker, ex-smoker, or current smoker), alcohol consumption (none, mild to moderate [<30 g/day], or heavy [≥30 g/day]), and intensity of regular exercise (low [<3 times with vigorous intensity or <5 times with moderate intensity/week] or high [≥3 times with vigorous intensity or ≥5 times with moderate intensity/week]) were collected. BMI was defined as weight/height^2^ (kg/m^2^). After overnight fasting over 8 h, blood sampling was conducted, and serum levels of glucose, GGT, total cholesterol, TG, high-density lipoprotein cholesterol (HDL), and low-density lipoprotein cholesterol (LDL) were assessed. FLI was calculated using BMI, WC, TG, and GGT [5]. Cumulative frequency of FLI ≥ 60 during 4 consecutive check-ups was assessed (range 0–4). FLI scores were established as follows: a score of 0 corresponded to FLI < 30, a score of 1 corresponded to 30 ≤ FLI < 60, and a score of 2 corresponded to FLI ≥ 60. By summing up FLI scores from four consecutive measurements, cumulative burden of fatty liver was quantified.

### 2.4. Statistical Analyses

Kidney cancer incidence was calculated as the number of events per person-time at risk (number of events per 1000 person-years). To examine statistical differences in baseline characteristics, Pearson χ^2^ test was applied. The Cox proportional hazards model was used to evaluate the hazard ratio (HR), 95% confidence interval (CI), and impact of cumulative fatty liver burden on kidney cancer. Adjustments were made for potential confounding factors, including smoking status, alcohol consumption, and physical activity. Model 1 was not adjusted, and model 2 was adjusted for smoking status, alcohol consumption, and physical activity. All statistical analyses were performed using SAS version 9.4 (SAS Institute, Cary, NC, USA) and R software version 4.3.1 (The R Foundation for Statistical Computing, Vienna, Austria, http://www.r-project.org (accessed on 16 June 2023)). Statistical significance was defined as a two-sided *p*-value less than 0.05.

## 3. Results

### 3.1. Baseline Characteristics of Study Population

A total of 1,007,906 subjects were included, 649 of which developed kidney cancer. The mean follow-up duration was 7.74 (±10.7) years. Table 1 presents the baseline characteristics of the subjects categorized according to cumulative frequency of FLI ≥ 60 during four consecutive check-ups. With an increase in cumulative frequency, there was a significant increase in the proportion of subjects who were current smokers and heavy drinkers. Additionally, BMI, WC, glucose levels, and lipid profiles (excluding HDL) showed an upward trend.

### 3.2. Cumulative Burden of NAFLD and Kidney Cancer

Model 1 was non-adjusted, and model 2 was adjusted for smoking, drinking, and exercise. Table 2 shows the hazard ratio of kidney cancer according to the mean FLI of four measurements. The HR (model 2) was 1.447 (95% CI: 1.26–1.853) for subjects with mean 30 ≤ FLI < 60. For those with a mean FLI ≥ 60, the HR was 2.391 (95% CI: 1.986–2.879). As the mean FLI increased, the HR for kidney cancer also increased.

Table 3 shows the incidence rates (IRs) and HRs according to a cumulative frequency of FLI ≥ 60. With increases in the cumulative frequency of FLI ≥ 60 (range, 0–4), both the IR and HR for kidney cancer rose. The HR for the population with 4 times of an FLI ≥ 60 had an HR of 2.541 (95% CI: 2.055–3.142) in model 2.

Table 4 shows the HR of kidney cancer according to FLI scores. With an increase in FLI scores, the IR and HR for kidney cancer showed an increasing trend. In model 2, subjects with an FLI score of 5 had an HR of 2.038 (95% CI: 1.478–2.811), and those with an FLI score of 8 had an HR of 3.143 (95% CI: 2.467–4.001) for kidney cancer.

### 3.3. Changes in NAFLD and Kidney Cancer

Table 5 shows the HRs for kidney cancer according to the FLI obtained at the first measurement and the FLI at the last measurement. The population with an FLI ≥ 60 at both the first measurement and the last measurement had the highest HR, i.e., 2.343 (95% CI: 2.008–2.941), in model 2. The group whose FLI was initially ≥ 60 but later decreased to below 60 showed a lower HR compared to the group that maintained an FLI ≥ 60 for both measurements (HR: 1.374 vs. 2.430). Furthermore, this group also had a lower HR than the group whose FLI was initially < 60 but subsequently increased to ≥60 (HR: 1.374 vs. 1.653).

## 4. Discussion

In this nationwide cohort study involving over a million men aged 20–39, the consistent association between NAFLD and an elevated risk of kidney cancer was a noteworthy finding. Importantly, this association remained robust across various lifestyle factors, such as smoking, alcohol consumption, and physical activity. The identification of NAFLD as an independent and modifiable risk factor for kidney cancer in young men presents a novel contribution to the field. Our results imply that addressing NAFLD could be a strategic approach for reducing the incidence of kidney cancer, especially in this demographic.

Various emerging therapeutics, including immune-oncologic drugs and target agents, have been used to treat kidney cancer, placing a significant economic burden on patients and society. The estimated annual cost for managing metastatic kidney cancer is approximately $ 1.6 billion [9].

Kidney cancer is closely associated with factors such as a high body mass index (BMI), smoking, hypertension (HTN), and chronic kidney disease [10]. A meta-analysis of 18 prospective studies identified a positive correlation between HTN and kidney cancer risk [11]. Obesity and smoking are known to elevate both the incidence and mortality rates of kidney cancer. A chronic inflammatory state of insulin resistance has been considered a key underlying mechanism. These factors tend to have more of an effect on young subjects than on older subjects [10,12]. A positive relationship between kidney cancer and overweight during childhood or adolescence has been reported in several studies. A Swedish study using data from the Swedish Cancer Registry reported a 6% increase in the risk of renal cell carcinoma (RCC) with each unit increase in BMI during adolescence [13]. A large prospective cohort study in the United States examined BMI changes, showing that weight gain in early (ages 18–35) and mid-adulthood (ages 35–50) is strongly associated with RCC risk, while weight gain after age 50 is not [10]. The incidence and mortality of kidney cancer vary significantly by region, with higher rates in developed countries [14]. Kidney cancer incidence is about twice as high in men compared to that for women. In young women, the impact of metabolic dysfunction on kidney cancer is blurred due to a protective effect of estrogen. Our previous study reported the same result for young women [1]. Thus, females were not included in the present study.

Metabolic-dysfunction-associated fatty liver disease has been identified as a risk factor for all-cause mortality and cardiovascular death [15]. Associations between NAFLD and various cancers have been reported in several studies. Liu et al. found an association between metabolic-dysfunction-associated liver disease and the risk of several cancers, including liver, kidney, and thyroid cancer, using the UK biobank resource [2]. Choi et al. revealed a higher risk of prostate cancer in non-obese people even in the absence of obesity or metabolic syndrome. A high FLI, an indicator of NAFLD, could predict breast cancer in postmenopausal women [4]. NAFLD is associated with an increased risk of digestive tract cancer, including stomach, colorectal, hepatic, biliary, and pancreatic cancer, in young adults [16]. NAFLD can be regarded as a metabolic disorder, particularly characterized by hepatic steatosis. The chronic inflammatory state associated with insulin resistance is a key mechanism underlying the increased incidence of cancer in patients with metabolic disorders. Several studies have demonstrated that ectopic hepatic fat promotes both local and systemic inflammation, insulin resistance, and metabolic dysfunction, all of which may facilitate cancer development [17]. PNPLA3, which encodes adiponutrin predominantly expressed in the liver and adipose tissue, has been implicated in the pathogenesis of fatty liver disease [18]. Notably, its common variant, p.I148M, has shown a strong association with susceptibility to fatty liver disease [19,20]. Furthermore, recent evidence suggests that the PNPLA3 p.I148M variant may also play a role in the development of liver and kidney cancer [2].

To our knowledge, this is the first study about the cumulative burden of NAFLD in kidney cancer. This study showed a decreased risk of kidney cancer in a population with improved NAFLD compared to those with persistent or aggravated NAFLD. This insight contributes to the current approach to cancer prevention. Young populations are showing little interest in fatty liver disease. However, the prevalence of NAFLD in young men has recently increased [21]. Considering this disease’s increasing prevalence, the economic burden of kidney cancer, and the significant impact of metabolic components at a young age, NAFLD in the young age group should be a topic of concern.

This study has several limitations. NAFLD was diagnosed using FLI values instead of a liver biopsy or ultrasonography. While liver biopsies and ultrasonography are considered gold-standard diagnostic tools, they are not applicable in population-based studies. However, the accuracy of the FLI has been extensively verified. esent study. Due to the nature of a longitudinal study, causal inferences between NAFLD and kidney cancer could not be established. Information on pathologic classifications, the disease burden of kidney cancer, or cancer-related mortality was not available. Additionally, the cohort consisted primarily of Korean participants, so validation using other populations is necessary. Despite these limitations, this study is notable as a population-based investigation into the cumulative impact of NAFLD on kidney cancer risk in young men. NAFLD might be an independent and modifiable risk factor for kidney cancer in this group. The findings of this study suggest that public health efforts should target young populations to help reduce the risk of kidney cancer. NAFLD should be included in such initiatives. Our findings suggest that we have a crucial opportunity to reduce premature morbidity and mortality associated with young-onset kidney cancer in the next generation.

## Figures and Tables

**Table 1:** Baseline characteristics.

*n* (%)		Cumulative Frequency of Fatty Liver Index (FLI) ≥ 60 During 4 Consecutive Check-Ups					
		0	1	2	3	4	*p*
		723,600	86,646	58,010	53,589	86,061	
Smoker	Non-	241,154 (33.33)	21,322 (24.61)	13,124 (22.62)	11,613 (21.67)	16,398 (19.05)	<0.0001
	Ex-	138,444 (19.13)	17,772 (20.51)	11,656 (20.09)	10,298 (19.22)	14,891 (17.3)	
	Current	344,002 (47.54)	47,552 (54.88)	33,230 (57.28)	31,678 (59.11)	54,772 (63.64)	
Alcohol consumption	No	186,117 (25.72)	16,783 (19.37)	10,683 (18.42)	9633 (17.98)	14,981 (17.41)	<0.0001
	Mild	468,250 (64.71)	55,822 (64.43)	37,035 (63.84)	34,039 (63.52)	53,410 (62.06)	
	Heavy	69,233 (9.57)	14,041 (16.21)	10,292 (17.74)	9917 (18.51)	17,670 (20.53)	
Regular exercise (high-intensity)		138,651 (19.16)	16,851 (19.45)	11,215 (19.33)	10,380 (19.37)	14,834 (17.24)	<0.0001
BMI, kg/m^2^		23.09 ± 2.45	26.09 ± 2.25	27.02 ± 2.39	27.93 ± 2.59	29.94 ± 3.3	<0.0001
WC, cm		79.87 ± 6.37	87.05 ± 5.98	89.15 ± 6.32	91.08 ± 6.73	95.64 ± 8.07	<0.0001
Fasting glucose, mg/dL		91.51 ± 12.94	95.05 ± 17.53	96.51 ± 19.51	98.3 ± 22.61	103.35 ± 30.23	<0.0001
Total cholesterol, mg/dL		186.73 ± 31.8	202.87 ± 33.99	206.32 ± 35.02	209.25 ± 36.05	214.28 ± 37.43	<0.0001
HDL, mg/dL		54.63 ± 17.61	49.66 ± 15.48	48.74 ± 16.3	47.74 ± 14.39	46.38 ± 14.98	<0.0001
LDL, mg/dL		109.63 ± 30.9	117.58 ± 35.51	119.15 ± 36.34	120.23 ± 37.58	120.97 ± 40.11	<0.0001
Triglyceride, mg/dL		102.41 (102.29–102.53)	167.11 (166.52–167.7)	182.52 (181.76–183.29)	198.04 (197.18–198.91)	233.83 (233.05–234.62)	<0.0001

BMI: body mass index; WC: waist circumference; HDL: high-density lipoprotein; LDL: low-density lipoprotein.

**Table 2:** Kidney cancer incidence based on mean fatty liver index (FLI).

FLI	*n*	Event	IR, per 1000 PY	HR (95% CI)	
				Model 1	Model 2
<30	535,979	237	0.06566	1 (Ref.)	1 (Ref.)
30–60	268,573	183	0.10054	1.528 (1.260, 1.853)	1.447 (1.191, 1.757)
≥60	203,354	229	0.16759	2.559 (2.134, 3.069)	2.391 (1.986, 2.879)
*p*-value				<0.0001	<0.0001

FLI: fatty liver index; IR: incidence rate; PY: person-year; HR: hazard ratio; CI: confidence interval.

**Table 3:** Kidney cancer incidence based on cumulative frequency of fatty liver index (FLI) > 60 over 4 consecutive check-ups.

	*n*	Event	IR, per 1000 PY	HR (95% CI)	
				Model 1	Model 2
0	723,600	354	0.0726	1 (Ref.)	1 (Ref.)
1	86,646	60	0.10261	1.414 (1.075, 1.858)	1.353 (1.028, 1.781)
2	58,010	60	0.15339	2.114 (1.608, 2.780)	2.006 (1.524, 2.642)
3	53,589	57	0.15767	2.173 (1.643, 2.875)	2.029 (1.531, 2.689)
4	86,061	118	0.2025	2.788 (2.264, 3.434)	2.541 (2.055, 3.142)
*p*-value				<0.0001	<0.0001

IR: incidence rate; PY: person-year; HR: hazard ratio; CI: confidence interval.

**Table 4:** Kidney cancer incidence based on cumulative fatty liver index (FLI) score.

FLI Score	*n*	Event	IR, per 1000 PY	HR (95% CI)	
				Model 1	Model 2
0	421,811	164	0.05793	1 (Ref.)	1 (Ref.)
1	109,655	61	0.08278	1.429 (1.065, 1.917)	1.394 (1.038, 1.871)
2	83,075	38	0.06776	1.168 (0.820, 1.662)	1.120 (0.786, 1.595)
3	76,315	54	0.10447	1.798 (1.322, 2.445)	1.693 (1.244, 2.306)
4	75,577	63	0.12222	2.097 (1.568, 2.804)	1.930 (1.440, 2.585)
5	56,192	49	0.1284	2.207 (1.604, 3.037)	2.038 (1.478, 2.811)
6	49,218	47	0.14109	2.430 (1.757, 3.361)	2.247 (1.621, 3.115)
7	50,002	55	0.16284	2.807 (2.068, 3.810)	2.575 (1.892, 3.505)
8	86,061	118	0.2025	3.489 (2.754, 4.420)	3.143 (2.467, 4.004)
*p*-value				<0.0001	<0.0001

FLI: fatty liver index; IR: incidence rate; PY: person-year; HR: hazard ratio; CI: confidence interval.

**Table 5:** Kidney cancer incidence based on fatty liver index (FLI) at initial and final measurements.

Initial FLI/Last FLI	*n*	Event	IR, per 1000 PY	HR (95% CI)	
				Model 1	Model 2
<60/<60	768,443	391	0.07547	1 (Ref.)	1 (Ref.)
<60/≥60	91,112	76	0.12553	1.676 (1.311, 2.143)	1.653 (1.291, 2.117)
≥60/<60	36,109	29	0.1165	1.529 (1.049, 2.230)	1.374 (0.941, 2.007)
≥60/≥60	112,242	153	0.20105	2.662 (2.208, 3.209)	2.430 (2.008, 2.941)
*p*-value				<0.0001	<0.0001

FLI: fatty liver index; IR: incidence rate; PY: person-year; HR: hazard ratio; CI: confidence interval.

## Data Availability

The Korean National Health Insurance Corporation (NHIC) provides researchers who meet specific criteria with access to confidential data through their website. These data are considered third-party, and we did not have any special privileges. Any researcher can submit a research proposal online (https://nhiss.nhis.or.kr/bd/ab/bdaba021eng.do (accessed on 16 June 2023)). If approved by the NHIC evaluation committee, researchers can access the de-identified NHIC dataset after paying a fee.
